# Smoking, drinking and body weight after re-employment: does unemployment experience and compensation make a difference?

**DOI:** 10.1186/1471-2458-9-77

**Published:** 2009-03-06

**Authors:** Kelly L Bolton, Eunice Rodriguez

**Affiliations:** 1 Department of Medicine, David Geffen School of Medicine, University of California at Los Angeles, Los Angeles, USA; 2 Department of Public Health and Primary Care, University of Cambridge, Cambridge, UK; 3 Department of Pediatrics and Center for Education in Family and Community, Stanford University School of Medicine, Palo Alto, USA

## Abstract

**Background:**

The impact of unemployment on behaviours such as smoking, drinking and body weight has been extensively researched. However, little is known about the possible protective effects of social assistance programs on these behavioural changes. This study examines the impact of unemployment periods on smoking, drinking and body weight changes among re-employed individuals and investigates whether the receipt of unemployment benefits influences these behaviours.

**Methods:**

This study used panel data provided by the Panel Study of Income Dynamics. Logistic regression models were used to analyze whether a period of unemployment in 2000 resulted in an increase in smoking and drinking or fluctuations in body weight among 2001 re-employed individuals in comparison with 1999 baseline levels. A total of 3,451 respondents who had been initially healthy and who had been continuously employed between 1998 and 1999 were included in the analysis.

**Results:**

Compared to stably employed respondents, those who had experienced periods of unemployment in 2000 and did not receive unemployment benefits were more likely than continuously employed individuals to report an increase in alcohol consumption (OR 1.8, 95% CI 1.0–3.1) and a decrease in body weight (OR 1.7, 95% CI 1.1–2.8) when they were already re-employed in 2001.

**Conclusion:**

Our findings suggest that the receipt of unemployment benefits confers a protective effect on health behavioural changes following periods of unemployment. These findings underscore the need to monitor the impact of unemployment assistance programs on health, particularly in light of the rapidly changing structure of employment and unemployment benefits.

## Background

A wealth of research about the health effects of economic insecurity has provided important evidence about the impact of unemployment on mental and physical well-being [1-11]. Yet, investigations into the short and long-term effects of unemployment on health behaviours such as smoking, drinking and body weight have yielded mixed results. It is increasingly being recognized that the dichotomous categories of employed/unemployed inadequately explain the current unemployment situation; unemployment must be studied in the context of social assistance programs, temporary employment and other atypical employment situations [12-16]. Mixed findings regarding the impact of unemployment on health behaviours may partially be attributed to heterogeneity within unemployment groups defined by the receipt of unemployment benefits.

### Alcohol

Socio-environmental and stress-based theories of alcoholism propose that alcohol abuse and addiction develop as coping reactions to stressful socio-environmental conditions such as unemployment[17]. However, it is also known that risky behaviours, such as problem drinking, result in increased unemployment[18]. Thus, it is likely that both causation and selection effects are involved in the relationship between alcohol consumption and unemployment. Cross-sectional studies in Scotland[19] and France[20] have found an increased frequency of alcohol abuse among the unemployed compared to the employed. Longitudinal studies have yielded mixed results; studies in the US[21] and Sweden[22] suggest that unemployment may increase alcohol consumption, but British and Norwegian studies demonstrate no such effect [23-25]. There is some evidence that the relationship between unemployment and alcohol use may be time-dependent; a recent investigation suggested that short-term unemployment decreases alcohol use while longer unemployment increases it[26].

### Smoking

Cigarette smoking, like alcohol use, has been found to be associated with stressful socioeconomic conditions such as unemployment[27]. Cross-sectional studies generally show that unemployed people are more likely to smoke than employed people[28], although unemployed smokers sometimes report smoking fewer cigarettes a day than their employed counterparts[29]. Longitudinal studies on unemployment and smoking have presented mixed findings[24] and are not consistent across countries[30]. Yet, there is some evidence that job loss is a risk factor for increasing smoking and that these effects may be long lasting[31].

### Body Weight

There is evidence from longitudinal studies that unemployment may impact body weight; data from the British Regional Heart Study[24] showed that men who had experienced some unemployment during the study period were more likely than continuously employed men to either lose or gain more than 10% of their body weight. In the study of a factory closure in Michigan, job loss was shown to have an impact on body weight. Subjects who lost their job showed greater instability in their weight over the two years of observation, even after re-employment[32]. In a nationwide representative sample of Finnish subjects, overweight in women was associated with long-term unemployment[33]. Yet, the association between food insecurity and body mass index (BMI) has been found to be curvilinear (i.e., thin and overweight people display different behaviours from normal weight subjects while facing periods of economic and food insecurity)[34].

### Objectives

As is the case with any epidemiological research short of a clinical trial, it is virtually impossible to control for all of the variables that could determine both employment instability and health behaviours simultaneously. However, when facing contradictory evidence on the relationship between unemployment and health behaviours, it would be precipitous to conclude that differences are due simply to a better or worse adjustment for the effects of selection. A key factor in understanding these mixed findings could be the social context in which the unemployment occurs.

Systems of formal support and social benefits are known to help maintain the health status of individuals exposed to unemployment. Perceived health status has been shown to differ between groups of unemployed people defined by the types of benefits they receive[30,35] Mental health is also affected by social assistance programs with the receipt of benefits being associated with a reduction in depression symptoms[35].

Our aim is to examine the impact of unemployment on smoking, drinking and body weight changes among re-employed individuals. This study expands upon previous work by investigating whether unemployment compensation influences these behaviours. Receiving unemployment compensation may help alleviate the stress of unemployment and thereby reduce the negative behavioural effects for two reasons. One reason is because receiving benefits reduces the psychological stress associated with a sharp decline in income. The second, in line with research on the study of income inequality, social capital and health[36], is because unemployment compensation might act as a form of social support for the unemployed. While the benefits conferred in unemployment compensation are primarily financial, previous research has shown that the individual's perception of support, in addition to the extent of actual supportive behaviours, mediates its health protective effects[37]. Thus, by reducing the financial, psychological and sociological strains of unemployment, the receipt of unemployment compensation may help reduce the negative behavioural changes associated with job-instability.

## Methods

### Participants

Data for this study were provided from the Panel Study of Income Dynamics (PSID) conducted by the University of Michigan, Ann Arbor. The PSID is a longitudinal survey of a representative sample of US individuals and the families with whom they reside. The study emphasizes the dynamic aspects of economic and demographic behaviour but also contains information on mental and physical health. Beginning with a national sample of 4,800 families in 1968, the PSID has traced individuals from those original households and their offspring since that time. Because the original focus of the study was the dynamics of poverty, the 1968 sample included a disproportionately large number of low income households. To make the sample more representative of the population, additional families were added in 1995 and 1999 resulting in a sample size of 6,997 households in 1999. The PSID has a very high response rate ranging between 96.9% and 98.5%. Approximately 92% of the sample is interviewed by telephone each year; the remaining 8% of the sample is interviewed in person[38]. The household head, defined as the husband in a husband-wife pair or the primary wage earner, is most often the respondent.

### Measures

#### Unemployment

We define employment as working full-time (> = 40 hours/week). Unemployment is defined as not working while still actively looking for work. Individuals who reported that they did not have a job and were not looking for work were not considered to be unemployed. In order to reduce the influence of prior unemployment and increase the homogeneity of the study group, only heads of household who were employed continuously in 1998, 1999 and at the time of the survey in 2001 were included in the study group. A total of 3,451 respondents were used in the analysis. The subjects were divided into three groups: those who were employed continuously in 2000 (N = 3,321), those who experienced unemployment in 2000 and received unemployment compensation (N = 51) and those who experienced unemployment in 2000 and who did not receive unemployment compensation (N = 79). The latter two groups were compared to the continuously employed group to examine their relative risk for increasing their smoking and drinking and either increasing or decreasing their body weight.

Unemployment insurance (UI) is a US. federal-state system that provides partial, temporary wage replacement to eligible workers. It is the largest unemployment compensation program in the US. The eligibility for (UI) varies by state and a comprehensive review of its conditions can be found elsewhere[39]. Briefly, eligibility has two major components: a monetary standard and a non-monetary standard. The monetary eligibility criteria can exclude some low-wage workers and temporary workers and, as a result, many states have adopted an alternative eligibility standard based on hours worked[40]. The main purpose of the non-monetary standard is to ensure that individuals who quit their jobs voluntarily or are fired cannot collect UI. Additional standards ensure that workers are available for work, are actively seeking employment and have not unduly refused work. Nearly all wage and salary workers are covered by the unemployment insurance system; the main exceptions are individuals who are self-employed and seasonal workers[41]. The weekly benefit allowance provided by UI again varies by state, but in general the maximum amount workers can claim is between 50 and 70% of the their previous average weekly wage[41]. Most states provide benefits for up to 26 weeks for workers with substantial work experience[41].

#### Smoking, Alcohol and Body Weight

In the PSID, alcohol consumption is defined as the average number of drinks per day (none, less than one, 1–2, 3–4, or 5 or more per day) over the past year. Cigarette usage is defined as the average number of cigarettes smoked per day over the past year (1–100 cigarettes).

Because of the relatively small numbers of individuals in the two unemployment categories, a binary dependent variable was used as the outcome variable to increase the power of the analysis. We created binary variables to determine whether the number of cigarettes smoked per day, the number of alcoholic drinks drunk per day or body weight had either increased or decreased between 1999 and 2001. An increase was defined as a 2001 self-reported value of smoking, drinking and body weight greater than the self-reported value in 1999; a decrease was defined as the inverse. To facilitate the interpretation of results, every outcome was modelled separately. Individuals with missing data for smoking, drinking or body weight in 1999 or 2001 were removed from the analysis.

In the analysis of an increase in alcohol consumption, individuals who responded that they drank five or more drinks per day in 1999 (the highest category included in the survey) could not have answered drinking more in 2001 and were removed from the model.

To control for prior body weight in the analysis of an increase or a decrease of body weight, we created a variable calculating the individual's BMI in 1999 using the standard formula[42].

### Statistical Analysis

A logistic regression analysis was performed to investigate whether a period of unemployment in 2000 resulted in a change in smoking, drinking and body weight among re-employed people in 2001 in comparison with baseline levels recorded in 1999. All of our models adjust for potential confounders of the relationship between the likelihood of a change in smoking, drinking and body weight and unemployment status. These include: age, sex, race, education, health status, income, number of household members and marital status of the respondents. In addition, we created different variables to control for prior smoking, drinking and weight for each of the outcome variables as described above. The income variable used was the total household post-government income created for use in the Cross-National Equivalent File[38]. This represents the combined income after taxes and government transfers. Likelihood ratio tests comparing models assuming the exposure showed a log-linear effect with a more general model including the exposure as a categorical variable were used to determine the most appropriate means for modelling, income, number of household members and, BMI in 1999. None of the likelihood ratio test statistics for these variables were significant for any of the outcome variables and in order to increase our power to detect an association, income, number of household members and BMI in 1999 were modelled as continuous variables.

We calculated the mean increase in the number of cigarettes smoked per day between 1999 and 2001. We also calculated the mean increase and decrease in body weight (measured in both pounds and BMI) between 1999 and 2001. Because alcohol consumption was measured as a categorical variable, we were only able to determine if consumption had increased by 1, 2 or 3–4 categories (equivalent to increasing consumption by 1–2 drinks per day, 3–4 drinks per day or 5 or more drinks per day). For all three outcome variables, we investigated any differences in the magnitude of changes by employment status in 2000.

The statistical analysis was performed using binary logistic regression analysis with SPSS 11.5 V and SPSS 13.0. Correlation and colinearity analyses were performed to confirm the appropriateness of the models.

## Results

### Descriptive

Women account for 19% of the continuously employed household heads included in the study sample, but they represent 27% and 28% of those who experienced a period of unemployment in the year 2000 with and without receiving unemployment compensation respectively. As described in Table 1, the average age of the continuously employed was 43.4 years, 40.6 years for those who experienced a period of unemployment with unemployment compensation and 40.4 years for those who did not receive compensation. Respondents who experienced unemployment and received unemployment compensation in 2000 were unemployed for an average of 11.4 weeks, and those who did not receive compensation were unemployed for an average of 8.2 weeks. The number of weeks of unemployment was included in a preliminary analysis as a controlling variable, but it was not included in the final model because it did not reach a significant level with any of the variables of interest.

**Table 1:** Descriptive Sample Characteristics by 2000 Employment Status

	Continuously employed (N = 3,321)	Experienced unemployment with compensation (N = 51)	Experienced unemployment without Compensation (N = 79)
Mean Age in 2001 (Median, SD)	43.5 (43.0, 9.9)	40.6 (40.5, 9.5)	40.4 (40.0, 10.1)
			
Weeks unemployed in 2000, Mean (Median, SD)	0 (0, 0)	11.4 (8.0, 9.0)	8.2 (4.5, 8.2)
			
% Women	19.2%	27.1%	28.2%
			
%African American	25.6%	31.3%	34.6%
			
Individual labor earnings 1999: Mean	$45,319	$37,589	$27,980
(Median, SD)	(35,000, 45,692)	(29,368, 30,832)	(22,750, 20,581)
			
Hours worked in 1999: Mean	2,327	2,259	2,139
			
1999 Baseline Drinking, Smoking and Weight:*			
% Did not drink alcohol in 1999	33.%	33%	30%
Mean drinking (if drinker) category (Median, SD)	1.4 (1, 0.6)	1.3 (1, 0.6)	1.7 (2, 0.5)
% Did not smoke in 1999	78%	78%	74%
Mean #cigarettes (if smoker) per day (Median, SD)	16.3 (15.0, 10.6)	16.3 (20.0, 10.1)	20.8 (20.0, 12.0)
Mean BMI in 1999 (Median, SD)	27.3 (26.6, 4.6)	27.3 (27.7, 4.7)	27.7 (26.6, 5.5)

SD = standard deviation. *None of the differences in drinking, smoking, and body weight were statistically significant in a GLM analysis controlling for age, gender and ethnicity

We investigated other possible differences among the three groups at baseline. Although the number of weeks and the hours per week worked in 1999 were similar, annual earnings were substantially different. The continuously employed in 2000 reported an average individual labour earning of $45,316 in 1999 (for a total of 2,115 hours of work), those who experienced unemployment in 2000 but received unemployment compensation reported an average earning of $37,589 in 1999 (for 2,259 hours of work) and those who experienced a period of unemployment in 2000 but did not receive unemployment compensation reported an average earning of $27,980 in 1999 (for 2,139 hours of work). The income difference between the groups was not statistically significant in a GLM analysis when controlling for the variables included in our analytical models.

The subjects who experienced unemployment in 2000 reported slightly higher levels of smoking, drinking and BMI in 1999 than those who were continuously employed in 2000. However, the difference was not significant when controlling for age, gender and ethnicity in a GLM analysis.

### Alcohol

Overall, 15.7% of respondents reported an increase in alcohol consumption between 1999 and 2001. Table 2 presents the results of binary logistic regression analysis to assess the impact of unemployment in the year 2000 and receipt of unemployment compensation on the likelihood of increasing alcohol consumption between the years 1999 and 2001. Prior history of alcohol consumption appeared to have the greatest effect on whether the number of alcoholic beverages consumed per day increased between 1999 and 2001. The higher the level of consumption in 1999, the less likely subjects were to increase their consumption in 2001. Respondents who experienced unemployment without receiving benefits had a greater likelihood than those continuously employed to increase their alcohol consumption (odds ratio = 1.76, 95% CI: 1.01 to 3.07). Women were less likely than men to increase their consumption. Non-married and separated individuals were more likely to increase their drinking than married individuals. Respondents with more than a high school education were less likely to increase their drinking, as were older respondents.

**Table 2:** Likelihood of Increasing Alcohol drinking between 1999 and 2001

	Frequency	Sig.	OR	95.0% C.I.	
	Total = 3,330			Lower	Upper
Employment status in 2000					
Continuously employed	3,204		1.00		
Unemployed with compensation	48	.91	1.05	.47	2.31
Unemployed without compensation	78	.04	1.76	1.01	3.07
					
Alcohol use in 1999					
0 drinks per day	1,100		1.00		
Less than 1 drink per day	1,703	<.001	.41	.33	.50
1–2 drinks per day	394	<.001	.28	.19	.41
3 or more drinks per day	133	<.001	.23	.12	.44
					
Education					
Less than high school	210		1.00		
High school	947	.04	.66	.45	.98
More than high school	1,358	.01	.58	.40	.86
Missing	815	.22	.78	.52	1.16
					
Marital status					
Married or partner	2,212		1.00		
Single/living alone	419	.01	1.70	1.16	2.48
Widowed, sep. or divorced	699	.04	1.41	1.01	1.95
					
Income level*	3,330	.15	1.04	.99	1.09
					
# household members	3,330	.09	.93	.86	1.01
					
Female	652	<.001	.49	.35	.68
					
Age	3,330	.11	.99	.98	1.00
					
Race					
White	2,196		1.00		
African American	861	.58	.93	.73	1.19
Other	273	.79	1.05	.72	1.52
					
Health status					
Excellent	885		1.00		
Very good	1,255	.11	1.22	.95	1.57
Good	942	.21	1.19	.90	1.56
Fair/Poor	248	.94	1.02	.67	1.55

* The income variable used was the total household post-government income created for use in the Cross-National Equivalent File[38]. This represents the combined income after taxes and government transfers. A Box-Cox transformation was performed on this variable to normalize it.

Among the 604 participants who reported an increase in alcohol consumption, 88.6% increased by 1–2 drinks per day, 8.9% increased by 3–4 drinks per day and 2.5% increased by at least 5 drinks per day. The proportion of individuals increasing their drinking by more than 3 drinks per day was slightly greater among those unemployed in 2000; among those employed, 11.2% increased by 3 or more drinks per day compared to 12.5% and 16.7% of individuals who were unemployed with and without compensation, respectively. The magnitude of the increase in alcohol consumption was not seen to differ greatly between those who were light and moderate/heavy drinkers at baseline in 1999.

### Smoking

In the study sample, 7.8% of participants reported an increase in smoking between 1999 and 2001. In logistic regression analysis, individuals who smoked in 1999 were significantly more likely to have increased their smoking in 2001 compared to individuals who reported smoking zero cigarettes per day in 1999 (Table 3). Employment status in 2000 also had an effect on smoking; both groups of unemployed individuals in 2000 were twice as likely to increase their smoking by 2001 compared to individuals who were continuously employed in 2000, but this was not statistically significant. Age had an effect on smoking changes over time, with older people being less likely to increase their smoking between 1999 and 2001.

**Table 3:** Likelihood of Increasing Tobacco Smoking between 1999 and 2001

	Frequency	Sig.	OR	95.0% C.I.	
	Total = 3,331			Lower	Upper
Employment status in 2000					
Continuously employed	3,205		1.00		
Unemployed with compensation	48	.06	2.23	.95	5.20
Unemployed without compensation	78	.09	1.90	.90	4.01
					
Cigarette use in 1999					
0 per day	2,606		1.00		
1–10 per day	303	<.001	15.52	11.12	21.67
11–20 per day	306	<.001	5.08	3.43	7.53
21 or more per day	116	<.001	4.08	2.24	7.44
					
Education					
Less than high school	210		1.00		
High School	947	.74	.91	.54	1.55
More than high school	1,359	.67	.89	.51	1.53
Missing	815	.87	1.05	.60	1.82
					
Marital status					
Married or partner	2,213		1.00		
Single living alone	419	.63	1.14	.67	1.94
Widowed, sep. or divorced	699	.06	1.52	.98	2.37
					
Income level*	3,331	.99	1.00	.93	1.07
					
# household members	3,331	.04	.88	.78	.99
					
Female	652	.47	.86	.56	1.31
					
Age	3,331	.001	.97	.96	.99
					
Race					
White	2,197		1.00		
African American	861	.29	.83	.58	1.17
Other	273	.48	1.22	.70	2.11
					
Health Status					
Excellent	885		1.00		
Very good	1,255	.75	1.06	.73	1.56
Good	943	.17	1.37	.89	1.98
Fair/Poor	248	.56	1.19	.66	2.14

* The income variable used was the total household post-government income created for use in the Cross-National Equivalent File[38]. This represents the combined income after taxes and government transfers. A Box-Cox transformation was performed on this variable to normalize it.

Among those who increased smoking, the average increase was by 8.5 cigarettes per day (95% CI 7.7–9.4). The average increase was slightly higher among those who were unemployed in 2000 and did not receive compensation. Individuals who experienced unemployment without compensation had an average increase of 9.5 cigarettes per day (95% CI 5.9–13.0). Those who were employed had an average increase of 8.4 (95% CI 7.4–9.3) and those who were unemployed with compensation had an average increase of 5.1 (95% CI 2.3–8.0).

### Body Weight

Overall, 30.0% of participants reported a decrease in body weight between 1999 and 2001, 47.6% reported an increase and 22.7% reported no change. As reported in Table 4, unemployed respondents who did not receive unemployment compensation were significantly more likely to lose weight than those who were continuously employed. Individuals who were unemployed and received unemployment benefits showed no significant difference. Women were less likely to decrease their weight than men. Age also had an effect, with older groups increasing their probability of decreasing weight. Baseline BMI was significant: those with a higher BMI were more likely to experience a decrease in their body weight. Respondents living in households with more members were less likely to decrease their body weight.

**Table 4:** Likelihood of Increasing or Decreasing Weight between 1999 and 2001

a: Likelihood of Increasing Weight between 1999 and 2001					
	Frequency	Sig.	OR	95.0% C.I.	
				
	Total = 3,331			Lower	Upper

Employment status in 2000					
Continuously employed	3,205		1		
Unemployed with compensation	48	0.77	0.9	0.51	1.64
Unemployed without compensation	78	0.25	0.8	0.48	1.21

BMI in 1999	3,331	<.001	1	0.94	0.97

Education					
Less than high school	210		1		
High School	947	0.73	1	0.7	1.29
More than high school	1,359	0.77	1.1	0.77	1.42
Missing	815	0.69	0.9	0.68	1.29

Marital status					
Married or partner	2,213		1		
Single living alone	419	0.97	1	0.75	1.34
Widowed, sep. or divorced	699	0.75	1	0.75	1.23

Income level*	3,331	0.46	1	0.98	1.05

# household members	3,331	0.25	1	0.98	1.09

Female	652	0.06	1.3	0.99	1.6

Age	3,331	0.03	1	0.98	1

Race					
White	2,197	.	1		
African American	861	0.02	1.2	1.04	1.47
Other	273	0.72	1.1	0.8	1.39

Health Status					
Excellent	885		1		
Very good	1,255	0.38	1.1	0.91	1.29
Good	943	0.15	1.2	0.95	1.4
Fair/Poor	248	0.02	1.4	1.04	1.9

b: Likelihood of Decreasing Weight between 1999 and 2001					

	Frequency	Sig.	OR	95.0% C.I.	
				
	Total = 3,331			Lower	Upper

Employment status in 2000					
Continuously employed	3,205	.	1		
Unemployed with compensation	48	0.4	1.3	0.71	2.38
Unemployed without compensation	78	0.02	1.7	1.09	2.76

BMI in 1999	3,331	<.001	1.1	1.05	1.09

Education					
Less than high school	210		1		
High School	947	0.27	0.8	0.6	1.15
More than high school	1,359	0.14	0.8	0.57	1.09
Missing	815	0.94	1	0.7	1.38

Marital status					
Married or partner	2,213		1		
Single living alone	419	0.72	0.9	0.69	1.3
Widowed, sep. or divorced	699	0.28	1.2	0.89	1.51

Income level*	3,331	0.6	1	0.95	1.03

# household members	3,331	0.05	0.9	0.88	1

Female	652	0.02	0.7	0.56	0.95

Age	3,331	0.04	1	1	1.02

Race					
White	2,197		1		
African American	861	0.48	1.1	0.88	1.3
Other	273	0.15	1.2	0.93	1.67

Health Status					
Excellent	885		1		
Very good	1,255	0.1	0.9	0.7	1.03
Good	943	0.44	0.9	0.74	1.14
Fair/Poor	248	0.2	0.8	0.58	1.12

* The income variable used was the total household post-government income created for use in the Cross-National Equivalent File[38]. This represents the combined income after taxes and government transfers. A Box-Cox transformation was performed on this variable to normalize it.

Employment status in the year 2000 did not significantly affect the risk of increasing body weight. Reporting fair or poor health significantly increased the risk of reporting body weight increases. African Americans were also at a significantly greater risk of increasing their body weight than were whites.

Among those whose weight decreased, the mean weight-loss was 7.1 pounds (95% CI 6.6–7.6) or 0.9 BMI units (95% CI 0.8–1.0). The mean decrease in body weight did not greatly differ according to employment status in 2000; in the employed group, the mean decrease was 6.9 pounds (95% CI 6.4–7.4) while in the unemployed groups the mean decrease was 8.0 (95% CI 4.2–11.7) and 8.3 (95% CI 5.2–11.5) for those with and without benefits, respectively. Among those who increased weight, the mean increase was by 12.8 pounds (95% CI 12.2–13.4) or 1.8 BMI units (95% CI 1.74–1.94). For those employed in 2000, the mean increase was 12.6 pounds (95% CI 11.9–13.2). For those unemployed with compensation the mean increase was 11.5 pounds (95% CI 8.5–14.6) and among those without compensation the mean increase was 14.8 (95% CI 8.2–21.6). The mean increase and decrease in BMI when stratified by employment status showed a similar pattern to weight change in pounds.

We used multiple regression models to investigate possible differences among the employment groups of interest and did not find statistically significant differences in the percentage of body weight change.

## Discussion

Our main findings are that employment and benefit status had an effect on the likelihood of changes in drinking and body weight. Those who experienced unemployment in the year 2000 and did not receive unemployment compensation were more likely to increase daily alcohol consumption by 2001 and decrease body weight. Those who experienced unemployment in 2000 were more likely to increase daily smoking by 2001 compared to the continuously employed but this was not statistically significant. We did not find evidence of large differences in the magnitude of smoking, drinking and body weight changes among employment groups. Among those who decreased body weight, the magnitude of weight-loss was similar in those who were unemployed without compensation and those who were continuously employed in 2000. Similarly, the magnitude of increases in alcohol consumption did not greatly differ between the two groups. Rather, a higher proportion of people who experienced unemployment and did not receive compensation reported weight loss or an increase in alcohol consumption compared to the continuously employed.

As drinking and smoking patterns show some degree of correlation, investigation into the impact of unemployment on increases in both smoking and alcohol consumption would be of use. However in this study, very few unemployed individuals increased both their smoking and alcohol consumption and such an analysis would be underpowered.

Although there is strong consensus in considering smoking increases as unhealthy behaviours that increase the risk of early death, we are more tentative in our interpretation of a decrease in body weight. A decrease in body weight is not a healthy sign necessarily because unintended body weight reductions could be a symptom of ill-health, and it is unknown if the observed weight drop was intentional or unintentional. Our findings are consistent with previous research reporting instability in body weight after unemployment, which may reflect a stress reaction. While the association between unemployment compensation and body weight change did not persist when body weight changes were restricted to a change of >5%, we think it useful to report even small changes since modest fluctuations in weight (such as the mean 7 pound weight-loss found in our study) are known to be associated with stress[43].

Moderate increases in alcohol consumption, as seen in our study, cannot be uniformly interpreted as detrimental to health. Studies generally report a J-shaped relationship between alcohol use and risk of total mortality with light to moderate drinkers showing a decreased risk compared to abstainers and heavy drinkers[44]. A health-protective effect of alcohol is generally defined as one or two drinks per day for men and one drink per day for women, although some disagreement still exists regarding the cut-offs for healthy drinking[45]. We explored the possibility of limiting our analysis to increases which resulted in unhealthy drinking in 2001 (alcohol consumption of at least 3 drinks a day for men and at least 2 drinks a day for women). However, the number of respondents available was too small and the data available precluded further investigation due to lack of power. While the higher likelihood of increasing drinking following a period of unemployment without compensation may not translate into an increased risk of poor health outcomes for all members of this group in the short term, we do find our results of concern as it is not possible to predict in which individuals modest increases in drinking will eventually lead to unhealthy drinking patterns[46]. Our finding that job loss increases the long-term risk of increasing alcohol consumption is consistent with previous literature showing an association between unemployment and an increase in alcohol consumption [21,22] but differs from other studies that report no such association [23-25]. This discrepancy could be due to differences in selection, but could also be due to differences in the social context of unemployment, particularly when comparing this study to studies performed in other countries. Our results indicate that those who experience periods of unemployment are more vulnerable and at a higher risk of adapting potentially unhealthy behaviours than the continuously employed. The difference was especially pronounced for those who did not receive unemployment compensation, even though, on average, they experienced fewer weeks of unemployment than those who received benefits.

Overall, our findings suggest that higher levels of stress exist among those who experience unemployment without compensation. This is an area that has not been previously explored and we hope that our findings will lead to further exploration of the association between unemployment compensation and body weight, smoking and alcohol consumption.

This study has certain limitations. As in any self-reported panel data our results could be influenced by information bias, particularly related to the use of self-reported smoking, drinking and body weight. The impact of possible information bias on our results is difficult to predict but seems unlikely to fully explain our findings. Because the variables on self-reported smoking and drinking used in this study reflect usual consumption patterns over the past year, we were not able to capture binging patterns or other fluctuations in substance use. The PSID is an established survey with a high participation rate which decreases the likelihood of selection bias. We attempted to control for the impact of a wide variety of potential confounders in our models. However, as little is known regarding the relationship between unemployment compensation and lifestyle changes, we cannot completely rule out the possibility of residual confounding. In order to increase the generalizability of the PSID sample to the general US population, we replicated the analysis, weighting the cases by the 2001 longitudinal weight variable created by the PSID[38]. While the main results remained the same, the significance levels for the explanatory and control variables were higher. We attenuated the possible effects of reverse causation by using prospective longitudinal data. In order to reduce the effects of unknown individual characteristics that might predispose individuals to unemployment, we analyzed only heads of household who were employed in 1998, 1999 and 2001. Those restrictions limited our sample size to 3,451 respondents, of which 51 experienced unemployment and received unemployment compensation in 2000 and of which 79 were unemployed but did not receive compensation. Because of this small sample size, we were unable to provide precise estimates of the magnitude of changes in smoking and body weight seen in the unemployed groups and limited in our ability to study heavy drinking patterns. Despite these limitations, this study provides important evidence that clears the way for further investigations.

In our study population, only 38% of respondents who were unemployed in 2000 reported receiving unemployment compensation which is consistent with national averages for receipt of unemployment insurance [47]. Information on why the unemployed workers did not receive benefits is not available. Failure to meet income requirements was probably not a primary reason since the average earnings of those unemployed who did not receive benefits exceeds the minimum wage requirements for UI in most states[39]. Those who did not receive unemployment compensation could have been more likely to have voluntarily left their job than those who did receive benefits. While this could represent a possible source of heterogeneity between employment groups, we do not consider it a source of confounding in our results given that a predicable change in employment status due to voluntary resignation is likely less harmful than an uncontrollable loss of income[48].

It is estimated that that over half of workers who are eligible for unemployment insurance do not file for benefits and we think it unlikely that ineligibility completely explains why a large portion of our study population did not file for UI benefits[47,49]. A recent report by the United States Government Accountability Office found that the most important predictor of whether eligible workers filed for UI benefits was if they had received them in the past. The report concludes that "a worker's perception of UI when faced with unemployment is key to whether that will worker will ever use the program[47]." Perceptions of support are known to be crucial in determining if any beneficial health effects are achieved from systems of social support [50]. Thus, the same favourable perceptions of UI that leads workers to file for benefits may also be responsible for the beneficial health effects seen in our results. This study focuses on the US and it will be interesting to replicate it in different countries with different compensation schemes. Previous work looking at the impact of unemployment benefits on health status has shown remarkable similarities among the US and European countries with different compensation schemes such as those found in Germany and the UK[35].

## Conclusion

The stated goals of UI revolve around financial stabilization both for the individual worker and the overall economy. However, unemployment assistance programs should not only aim to reduce the financial impact but also the detrimental health effects of unemployment. This study suggests that UI may help to alleviate the drinking and body weight changes associated with employment instability. Recipiency rates for unemployment insurance have dropped steadily over the past 40 years, sparking recent legislative changes to make the UI program more responsive to the needs of US workers[41]. Continuing to develop an understanding of the effects of unemployment compensation on health will be increasingly important as rapid and substantial changes occur in the UI program and the overall structure of employment. Further research in this area is necessary to explore possible differential behavioural impacts of workers in different occupational categories and possible effects on concurrent policies geared toward decreasing tobacco and alcohol consumption in the general population.

## Competing interests

The authors declare that they have no competing interests.

## Authors' contributions

Both KLB and ER conceived of the design of study, participated in the statistical analysis and drafted the manuscript.

## Pre-publication history

The pre-publication history for this paper can be accessed here:


